# Antineuropathic Profile of *N*-Palmitoylethanolamine in a Rat Model of Oxaliplatin-Induced Neurotoxicity

**DOI:** 10.1371/journal.pone.0128080

**Published:** 2015-06-03

**Authors:** Lorenzo Di Cesare Mannelli, Alessandra Pacini, Francesca Corti, Serena Boccella, Livio Luongo, Emanuela Esposito, Salvatore Cuzzocrea, Sabatino Maione, Antonio Calignano, Carla Ghelardini

**Affiliations:** 1 Department of Neuroscience, Psychology, Drug Research and Child Health—Neurofarba—Pharmacology and Toxicology Section, University of Florence, Florence, Italy; 2 Department of Experimental and Clinical Medicine—DMSC—Anatomy and Histology Section, University of Florence, Florence, Italy; 3 Department of Experimental Medicine, Division of Pharmacology, The Second University of Naples, Naples, Italy; 4 Department of Biological and Environmental Sciences, University of Messina, Messina, Italy; 5 Young Against Pain (YAP) group, Italy; 6 Department of Pharmacy, University of Naples “Federico II”, Naples, Italy; University of Texas at Dallas, UNITED STATES

## Abstract

Neurotoxicity is a main side effect of the anticancer drug oxaliplatin. The development of a neuropathic syndrome impairs quality of life and potentially results in chemotherapy dose reductions and/or early discontinuation. In the complex pattern of molecular and morphological alterations induced by oxaliplatin in the nervous system, an important activation of glia has been preclinically evidenced. *N*-Palmitoylethanolamine (PEA) modulates glial cells and exerts antinociceptive effects in several animal models. In order to improve the therapeutic chances for chemotherapy-dependent neuropathy management, the role of PEA was investigated in a rat model of oxaliplatin-induced neuropathy (2.4 mg kg^-1^ daily, intraperitoneally). On day 21, a single administration of PEA (30 mg kg^-1^ i.p.) was able to reduce oxaliplatin-dependent pain induced by mechanical and thermal stimuli. The repeated treatment with PEA (30 mg kg^-1^ daily i.p. for 21 days, from the first oxaliplatin injection) prevented lowering of pain threshold as well as increased pain on suprathreshold stimulation. *Ex vivo *histological and molecular analysis of dorsal root ganglia, peripheral nerves and spinal cord highlighted neuroprotective effects and glia-activation prevention induced by PEA repeated administration. The protective effect of PEA resulted in the normalization of the electrophysiological activity of the spinal nociceptive neurons. Finally, PEA did not alter the oxaliplatin-induced mortality of the human colon cancer cell line HT-29. The efficacy of PEA in neuropathic pain control and in preventing nervous tissue alteration candidates this endogenous compound as disease modifying agent. These characteristics, joined to the safety profile, suggest the usefulness of PEA in chemotherapy-induced neuropathy.

## Introduction

Oxaliplatin is a chemotherapeutic agent effective in various solid tumours [1], [2], in particular, it was introduced for the management of the advanced stages of colorectal cancer. Currently, oxaliplatin is the regimen of choice for the adjuvant treatment of patients with curative resection of node-positive colon cancer [3], [4], [5]. The dose-limiting toxicity of this compound is the development of peripheral neuropathy with glove-and-stocking distribution sensory loss, combined with paresthesia, dysesthesia, and pain [6], [7]. Acute neurotoxicity of oxaliplatin commonly reverses within a week [8], [9], on the contrary a chronic neurological syndrome, related to the total cumulative dose as well as the dose-intensity of treatment [10] persists between and after treatments [11], [12], [13] negatively influencing patient’s quality of life.

There is no currently univocally-accepted proven therapy for oxaliplatin-induced neuropathy [11], [14]. Most randomized controlled trials testing a variety of drugs with diverse mechanisms of action failed to reveal an effective treatment [15], [16], [17]. Only symptomatic adjuvant compounds, like duloxetine, demonstrated clinical benefit [18]. Neuroprotective, safe, preventive agents as adjuvant to chemotherapy are a therapeutic need.

*N*-Palmitoylethanolamine (PEA), the endogenous amide between palmitic acid and ethanolamine, belongs to the family of fatty acid ethanolamides (FAEs), a class of lipid mediators. PEA exerts antinociceptive and anti-hyperalgesic effects in several animal models [19], [20]. Moreover, PEA protects nervous tissue in neuropathic conditions [21], prevents neurotoxicity and neurodegeneration [22], [23], and inhibits peripheral inflammation, mast cell degranulation [24] and glial cell activation [25]. Its efficacy and safety were shown in a variety of clinical trials focused on persistent pain treatment such as carpal tunnel syndrome, sciatic pain, low-back pain, osteoarthritis, failed back surgery syndrome, diabetic neuropathy, neuropathic pain in stroke and multiple sclerosis, chronic pelvic pain, and postherpetic neuralgia [26], [27], [28], [29]. Recently, the efficacy of PEA in chemotherapy-induced painful neuropathy was suggested since Truini et al. [30] demonstrated the efficacy of PEA in relieving pain and improving neurophysiological functions in patients undergoing thalidomide and bortezomib treatment.

This evidence prompted us to investigate the role of PEA treatment in oxaliplatin-induced neuropathic pain. The anti-neuropathic role of PEA was evaluated in oxaliplatin-treated animals by analyzing pain behavior in relation to molecular, morphological and functional protection of the nervous system.

## Materials and Methods

### Animals

For all the experiments described below, male Sprague-Dawley rats (Harlan, Varese, Italy) weighing approximately 200–250 g at the beginning of the experimental procedure, were used. Animals were housed in CeSAL (Centro Stabulazione Animali da Laboratorio, University of Florence) and used at least one week after their arrival. Four rats were housed per cage (size 26 × 41 cm); animals were fed a standard laboratory diet and tap water *ad libitum*, and kept at 23 ± 1°C with a 12 h light/dark cycle, light at 7 a.m. All animal manipulations were carried out according to the European Community guidelines for animal care (DL 116/92, application of the European Communities Council Directive of 24 November 1986 (86/609/EEC). The ethical policy of the University of Florence complies with the Guide for the Care and Use of Laboratory Animals of the US National Institutes of Health (NIH Publication No. 85–23, revised 1996; University of Florence assurance number: A5278-01). Formal approval to conduct the experiments described was obtained from the Animal Subjects Review Board of the University of Florence. Experiments involving animals have been reported according to ARRIVE guidelines [31]. All efforts were made to minimize animal suffering and to reduce the number of animals used.

### Oxaliplatin model and pharmacological treatments

Rats were treated with 2.4 mg kg^-1^ oxaliplatin (Sequoia Research Products, Pangbourne, UK), administered intraperitoneally (i.p.) for 5 consecutive days every week for 3 weeks (15 i.p. injections) [32]. Oxaliplatin was dissolved in a 5% glucose-water solution.

The model used for the present research is consistent with the clinical practice. 2.4 mg kg^-1^ oxaliplatin corresponds to the common human dosage (considering the Km factor 37 for the conversion of animal doses to the Human Equivalent Dose; [33], [34]. The daily repeated administration of 2.4 mg kg^-1^ performed in the animal model allows to obtain a cumulative dose of 36 mg kg^-1^ corresponding to 1332 mg/m^2^. This dosage mimics the clinical cumulative oxaliplatin dose causing chronic neuropathy. Moreover, in our condition the inorganic platinum plasmatic levels after 21 days of treatment is 3.573 ± 0.217 μg/mL in line to human plasma concentration [35].

Ultramicronized PEA (Epitech, Padova, Italy) was dissolved in PEG and Tween 80 2:1 (Sigma-Aldrich, Milan, Italy), and kept overnight under gentle agitation with a micro stirring bar. Before injection, sterile saline was added so that the final concentrations of PEG and Tween 80 were 20 and 10% v/v, respectively. PEA (10 or 30 mg kg^-1^) was administered acutely i.p. on day 21 or daily starting from the first day of oxaliplatin administration up to day 20. Control animals received equivalent volumes of vehicles. Behavioral tests were performed on day 21. Morphological and biochemical tests were performed on day 21.

### Paw-pressure test

The nociceptive threshold of rats was determined with an analgesimeter (Ugo Basile, Varese, Italy), according to the method described by Leighton et al. [36]. Briefly, a constantly increasing pressure was applied to a small area of the dorsal surface of the hind paw using a blunt conical probe by a mechanical device. Mechanical pressure was increased until vocalization or a withdrawal reflex occurred while rats were lightly restrained. Vocalization or withdrawal reflex thresholds were expressed in grams. Rats scoring below 40 g or over 75 g during the test before drug administration were rejected (25%). For analgesia measures, mechanical pressure application was stopped at 120 g.

### Von Frey test

The animals were placed in 20 cm × 20 cm Plexiglas boxes equipped with a metallic mesh floor, 20 cm above the bench. Animals were allowed to habituate themselves to their enviroment for 15 min before the test. An electronic Von Frey hair unit (Ugo Basile, Varese, Italy) was used: the withdrawal threshold was evaluated by applying forces ranging from 0 to 50 g with a 0.2 g accuracy. Punctuate stimulus was delivered to the mid-plantar area of each anterior paw from below the mesh floor through a plastic tip and the withdrawal threshold was automatically displayed on the screen. The paw sensitivity threshold was defined as the minimum force required to elicit a robust and immediate withdrawal reflex of the paw. Measurements were performed on the anterior paw since it shows the higher sensitivity to this test [37]. Voluntary movements associated with locomotion were not considered as a withdrawal response. Stimuli were applied to each anterior paw at 5 s intervals. Measurements were repeated 5 times and the final value was obtained by averaging the 5 measurements [38].

### Cold plate test

The animals were placed in a stainless box (12 cm × 20 cm × 10 cm) with a cold plate as floor. The temperature of the cold plate was kept constant at 4°C ± 1°C. Pain-related behaviors (i.e. lifting and licking of the hind paw) were observed and the time (s) of the first sign was recorded. The cut-off time of the latency of paw lifting or licking was set at 60 s.

### Rota-rod test

The Rota-rod apparatus (Ugo Basile, Varese, Italy) consisted of a base platform and a rotating rod with a diameter of 6 cm and a non-slippery surface. The rod was placed at a height of 25 cm from the base. The rod, 36 cm in length, was divided into 4 equal sections by 5 disks. Thus, up to 4 rats were tested simultaneously on the apparatus, with a rod-rotating speed of 10 r.p.m. The integrity of motor coordination was assessed on the basis of the time the animals kept their balance on the rotating rod up to a maximum of 10 min (600 s). The number of falls from the rod was also measured. After a maximum of 6 falls, the test was suspended and the time was recorded.

### Western blot evaluation

On day 21 sciatic nerves (1.5 cm segments were obtained at mid-thigh level approximately 1.0 cm proximal to the trifurcation), L4-L5 dorsal root ganglia (DRG) and the lumbar portion of the spinal cord were dissected, frozen using liquid nitrogen and then homogenized on ice in ice-cold hypotonic buffer A (10 mM Hepes pH 7.9, 10 mM KCl, 0.1 mM EDTA, 0.1 mM EGTA, 1 mM DTT, 0.5 mM PMSF with a protease inhibitor cocktail) using a Politron PT 13,000 D tissue homogenizer (Kinematica). After 15 min incubation on ice, the homogenates were centrifuged at 1,000 g for 10 min at 4°C. Supernatants containing cytoplasm extracts were stored at -80°C. Nuclear pellets were resuspended in ice-cold buffer B (1% Triton X-100, 150 mM NaCl, 10 mM TRIS-HCl pH 7.4, 1 mM EGTA, 1 mM EDTA, 0,2 mM PMSF, 20 mm leupeptin, 0,2 mM sodium orthovanadate) and the tubes were vigorously rocked at 4°C for 30 min on a shaking platform. The nuclear extracts were centrifuged at 13,000 g for 15 min at 4°C. The supernatants were frozen in aliquots at -80°C until use. Protein concentrations were determined by the Bradford method using bovine serum albumin (BSA) as standard. Proteins from cytoplasm and nuclear fraction were added to sample buffer [0.125 M Tris-HCl, (pH 6.8), 4% SDS, 20% glycerol, 10% β-mercaptoethanol, 0.004% bromphenol blue], and boiled in a water bath for 5 min. Protein samples (40 μg per lane) were separated on denaturing 12% SDS polyacrylamide gel and transferred to a nitrocellulose membrane. Non-specific binding to the membrane was blocked for 1 h at room temperature with 5% milk in PBS. Membranes were then incubated at room temperature with primary antibody in milk-PBS- Tween 20 0.1% (PMT) for IκB-α (1:1000; Santa Cruz Biotechnology) or for COX-2 (1:1500; Cayman Chemicals), washed three times with PBS -0.1% Tween, and then incubated for 1 h at room temperature with a secondary antibody (peroxidase-conjugated goat anti-rabbit IgG, 1:2000; Jackson ImmunoResearch, West Grove, PA). Polyclonal anti-actin antibody was used as an internal standard for cytoplasm.

Signals were detected with enhanced chemiluminescence (ECL) detection system reagent according to the manufacturer’s instructions (SuperSignal West Pico Chemiluminescent Substrate, Thermo Fisher Scientific, Waltham, MA, USA). The relative expression of the protein bands was quantified by densitometry with Gel Logic 2200 PRO software (Carestream Health, Rochester, NY, USA) and standardized to β-actin levels. Images of blot signals (8 bit/600 dpi resolution) were imported to analysis software (ImageQuant TL, v2003, Amersham Biosciences, Piscataway, NJ, USA). A preparation of commercially available molecular weight markers (Precision Plus Protein Standard, Bio-Rad, Hercules, CA, USA), consisting of proteins of molecular weight 10 to 250 kDa, was used to define molecular weight positions and as reference concentrations for each molecular weight.

### Histologic and morphometric studies on dorsal root ganglia

On day 21, L4-L5 DRGs were excised from rats of each group, and fixed by immersion in 4% neutral buffered formalin. The tissues were then washed with PBS, dehydrated with ascending grades of reagent alcohol, cleared in two changes of xylene and infiltrated with paraffin (Diapath, Milan, Italy). DRGs were sliced to 5 μm, mounted on charged slides and stained with Azan-Mallory method.

Cellular dimensions of L4-L5 DRGs were measured using a method adapted from Tomiwa et al. [39] and Coggeshall et al. [40] and following previously established size criteria [41]. In these sections, using a 100x oil immersion objective lens, the numbers of neurons with nuclei, nucleoli, multiple nucleoli, and nucleolar eccentricity were counted. The nucleolus was considered eccentric when its center (or that of the largest one if there appeared to be more than one) lay in the outer half of the radius of the nucleus. The results were expressed as percentage of those cells with a visible nucleolus. Four consecutive sections for each animal were analyzed. Soma areas were computed measuring between 50 and 100 cells for each animal from several sections. The reported data were obtained by averaging the data of L4 and L5 ganglia. DRG neurons with a soma area < 600 μm^2^ were classified as small, between 600 and 1200 μm^2^ as medium and > 1200 μm^2^ as large.

### Immunohistochemical evaluation of activating transcription factor 3 (ATF3) in the sciatic nerve and L4-L5 DRGs

On day 21, sciatic nerves and L4-L5 DRGs were rapidly dissected and paraffin-embedded. 10 μm DRGs sections and longitudinal sciatic nerve sections were obtained using a microtome and mounted on Superfrost Plus slides. Sectioned tissues were incubated for 1 h at room temperature in a blocking solution of 3% normal donkey serum in PBS with 0.3% Triton-X100 and then incubated overnight at 4°C in primary antisera against the activating transcription factor 3 (rabbit anti-ATF3, 1:500; Santa Cruz Biotechnology, USA). Finally the sections were washed in PBS and exposed to secondary antibodies and, in the sciatic nerve, visualized with VIP (Vector Laboratories, DBA Italia, Milan, Italy) in 0.1 M Tris buffer whereas in L4-L5 DRGs was revealed with 3,3V-diaminobenzidine tetrachloride (DAB). For quantification of the number of ATF3 positive cells within the sciatic nerve, a manual counting system was used as reported by Peters et al. [42]. Four optic fields on individual sections were assessed and the results were expressed as number of ATF3 positive profiles/mm^2^ (data not shown).

### Electrophysiological recordings

On day 21, rats were initially anaesthetized with sodium pentobarbital (50 mg/kg, i.p.). After tracheal cannulation, a catheter was placed into the right external jugular vein, to allow continuous infusion of propofol (5–10 mg/kg/h, i.v.) and spinal cord segments L4-L6 were exposed by laminectomy, medially near the dorsal root entry zone up to a depth of 1200 μm [43]. An elliptic rubber ring (about 3 x 5 mm) was tightly sealed with silicone gel onto the surface of the cord. This ring formed a trough with about 50 μl capacity over the spinal segments used for topical spinal drug application and to gain access to spinal neurons. Animals were then secured in a stereotaxic apparatus (David Kopf Instruments, Tujunga, CA, USA) supported by clamps attached to the vertebral processes on either side of the exposure site [44], [45]. Body temperature was maintained at 37°C with a temperature-controlled heating pad. A glass-insulated tungsten filament electrode (3–5 MΩ) (FHC Frederick Haer & Co., ME, USA) was used to record single unit extracellular activity of dorsal horn nociceptive-specific (NS) neurons. NS neurons were defined as those neurons responding only to high-intensity (noxious) stimulation [46]. For control and treated animals, each neuron was characterized by giving a mechanical stimulation to the injected paw by von Frey filament with a bending force of 5.8 N/20 mm^2^ (noxious stimulation) for 3 s with it slightly buckled [43] to confirm NS response patterns. Only neurons that specifically responded to noxious hind paw stimulation, without responding to stimulation of the surrounding skin/tissue, were considered for recordings. The recorded signals were amplified and displayed on a digital storage oscilloscope to ensure that the unit under study was unambiguously discriminated throughout the experiment. Signals were also fed into a window discriminator, whose output was processed by an interface CED 1401 (Cambridge Electronic Design Ltd., UK) connected to a Pentium III PC. Spike2 software (CED, version 4) was used to create peristimulus rate histograms on-line and to store and analyze digital records of single unit activity off-line. Configuration, shape, and height of the recorded action potentials were monitored and recorded continuously using a window discriminator and Spike2 software for on-line and off-line analysis. This study only included neurons whose spike configuration remained constant and could clearly be discriminated from activity in the background throughout the experiment, indicating that the activity from one neuron only and from the same neuron was measured. The neuronal activity was expressed Hz. At the end of the experiment, each animal was killed with a lethal dose of pentobarbital. Groups of 3–6 rats have been used and 2–3 NS neurons were recorded for each animal. The single extracellular recordings were performed on day 21 of treatment.

### Immunohistochemistry of spinal cord and brain glia

On day 21, rats were sacrificed, the L4/L5 segments of the spinal cord were exposed from the lumbovertebral column via laminectomy and identified by tracing the dorsal roots from their respective DRG. The brains were removed, sliced in coronal sections and areas of interest were identified using Paxinos and Watson’s atlas [47]. For all subsequent staining experiments, three sections from each brain corresponding to 3.5, 4.5, and 5.5 mm caudal to the bregma were selected for analysis.

Formalin-fixed cryostat sections (20 m) were incubated for 1 h in blocking solution (Bio-Optica, Milan, Italy) at room temperature; and were then incubated for 24 h at 4°C in PBST containing rabbit primary antisera diluted 1:1000 and 5% normal donkey serum. The primary antibody was directed against Iba1 (rabbit, 1:1000; Wako Chemicals, Richmond, USA) for microglial staining and against glial fibrillary acidic protein (GFAP; mouse, 1:5000; Chemicon, Temecula, USA) for astrocyte staining. After rinsing in PBST, sections were incubated in donkey anti-rabbit IgG secondary antibody labeled with Alexa Fluor 568 (1:1000, Invitrogen, Carlsbad, USA) at room temperature for 1 h.

Negative control sections (no exposure to the primary antisera) were processed concurrently with the other sections for all immunohistochemical studies. We obtained a single optical density value for the dorsal horns by averaging the two sides in each rat, and these values were compared to the homologous average values from the vehicle-treated animals.

Images were acquired by a motorized Leica DM6000B microscope equipped with a DFC350FX camera (Leica, Mannheim, Germany). Microglia and astrocyte morphology was assessed by inspection of at least three fields (40X 0.75NA objective) in the dorsal horn and cerebral areas *per* section.

Quantitative analysis of GFAP and Iba1-positive cells was performed by collecting at least three independent fields through a 20X 0.5NA objective. GFAP-positive cells were counted using the “cell counter” plugin of ImageJ, while Iba1-positive cells were quantified by means of the automatic thresholding and segmentation features of ImageJ. The GFAP signal in immunostained sections was quantified using FIJI software (distributed by ImageJ, NIH, Bethesda, Maryland, USA) by automatic thresholding images with the aid of the "Moments" algorithm, which we found to provide the most consistent pattern recognition across all acquired images. Results (not shown), given as the area fraction (%) occupied by the thresholded GFAP signal, revealed a common trend between GFAP expression and astrocyte cell number. Five spinal cord sections and 5 sections for each brain area were analyzed for each animal.

### Cell culture and treatments

The human colon cancer cell line HT-29 was obtained from American Type Culture Collection (Rockville, MD). HT-29 were cultured in DMEM high glucose with 20% FBS in 5% CO_2_ atmosphere at 37° C. Media contained 2 mM L-glutamine, 1% essential aminoacid mix, 100 IU ml^-1^ penicillin and 100 μg ml^-1^ streptomycin (Sigma, Milan, Italy). HT-29 cells were plated in 96-wells cell culture (1∙10^4^/well) plates, and after 48h they were treated with oxaliplatin (0–100 μM) for 24 or 48h. PEA (10 μM) was used in the presence of oxaliplatin for 24 or 48h. These concentrations were chosen according to previous published data [14], [48] and, as regards oxaliplatin, with plasmatic concentration of treated rats.

### Cell viability assay

HT-29 cell viability was evaluated by the reduction of 3-(4,5- dimethylthiozol-2-yl)-2,5-diphenyltetrazolium bromide (MTT) as an index of mitochondrial compartment functionality. Cells were plated into 96-well cell culture plates, and after 48h they were treated. Oxaliplatin, at various concentrations, was incubated in DMEM in the presence of 10 μM PEA for 48 h and 5 days. After extensive washing, 1 mg/ml MTT was added into each well and incubated for 30 minutes at 37°C. After washing, the formazan crystals were dissolved in 150 μl dimethyl sulfoxide. The absorbance was measured at 550 nm. Experiments were performed in quadruplicate on at least three different cell batches.

### Statistical analysis

Behavioral measurements were performed on 12 rats for each treatment carried out in 2 different experimental sets. For behavioral experiments standard ANOVA followed by Fisher’s protected least significant difference procedure were used. Repeated measures ANOVA followed by Fisher’s protected least significant difference procedure were used for behavioral experiments when two different time points were compared for the same group. For the immunoblot quantitation a and the electrophysiological measurements One-way ANOVA followed by Bonferroni post-test, for comparisons between groups were performed. Histological, morphometric and immunohistochemical analyses were performed on 6 rats per group, evaluating 6 sections each of sciatic nerve, L4-L5 DRG, spinal cord and S1 area for each animal. DRG values are reported as means of L4 and L5. One-way repeated measure ANOVA followed by the Mann–Whitney test was used. Data from cell culture measurements are expressed as mean ± SEM and analysis of variance (ANOVA) was performed; a Bonferroni’s significant difference procedure was used as post hoc comparison. All assessments were made by researchers blinded to cell or rat treatments. Data were analyzed using the ‘‘Origin 8.1” software (OriginLab, Northampton, USA). Differences were considered significant at a *P*<0.05.

## Results

### Effects of PEA on oxaliplatin-induced neuropathic pain

**Fig 1:**
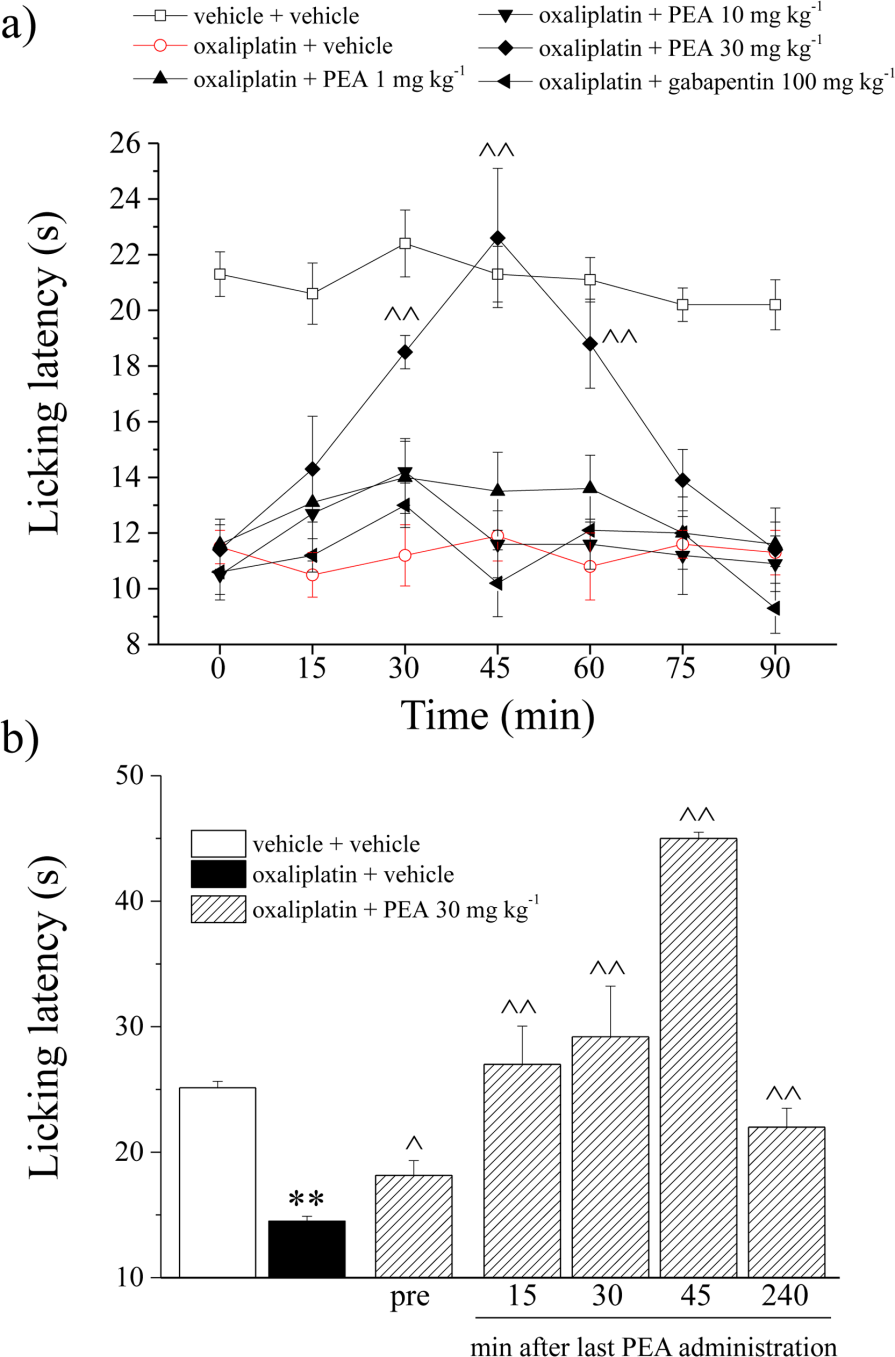
Behavioral measures. *Pain*: *thermal non-noxious stimuli*. The Cold plate test was used to evaluate the pain threshold measuring the latency to pain-related behavior (lifting or licking of the paw). a) Effect of PEA (1–30 mg kg^-1^ i.p.) after acute administration on day 21 of the oxaliplatin treatment (2.4 mg kg^-1^ oxaliplatin daily i.p.); b) Effect of PEA (30 mg kg^-1^ i.p.) after repeated administrations performed daily starting from the first day of oxaliplatin administration. Behavioral evaluations were performed on day 21, 24h after treatment (pre) and over time after a new injection. Control animals were treated with vehicles. Each value represents the mean of 12 rats per group, performed in two different experimental sets. **P<0.01 versus vehicle + vehicle; ^P<0.05 and ^^P<0.01 versus oxaliplatin + vehicle.

The daily treatment of rats with a clinically relevant dose of oxaliplatin (2.4 mg kg^-1^; [35]) induces an increasing painful condition [37]. On day 21, oxaliplatin caused a lowering of the threshold to cold stimuli which do not normally provoke pain (Cold plate test). The licking latency decreased from 21.3 ± 0.8 s (Fig 1A; vehicle + vehicle) to 11.5 ± 0.6 s (oxaliplatin + vehicle). Acute i.p. administration of PEA (30 mg kg^-1^) significantly relieved pain 30 min after administration. The effect lasted for 60 min (Fig 1A). The doses of 1 and 10 mg kg^-1^ were ineffective. As shown in the S1 Table, PEA (30 mg kg^-1^ i.p.) did not alter the normal pain threshold of vehicle-treated animals. The lack of antinociceptive properties in control rats was evaluated by thermal (Hot plate and Cold plate tests) and mechanical (Paw pressure) stimuli (S1 Table). The pain reliever effect of PEA (30 mg kg^-1^) was evaluated also after a daily repeated treatment starting from day 1 to day 20 of the oxaliplatin protocol. In Fig 1B is shown the sensitivity to a cold surface measured on day 21. Twenty-four hours after the last administration (pre), PEA-treated rats showed a pain threshold increased by about 40%. A further PEA injection fully reverted oxaliplatin-induced alteration peaking 45 min after treatment (Fig 1B).

**Fig 2:**
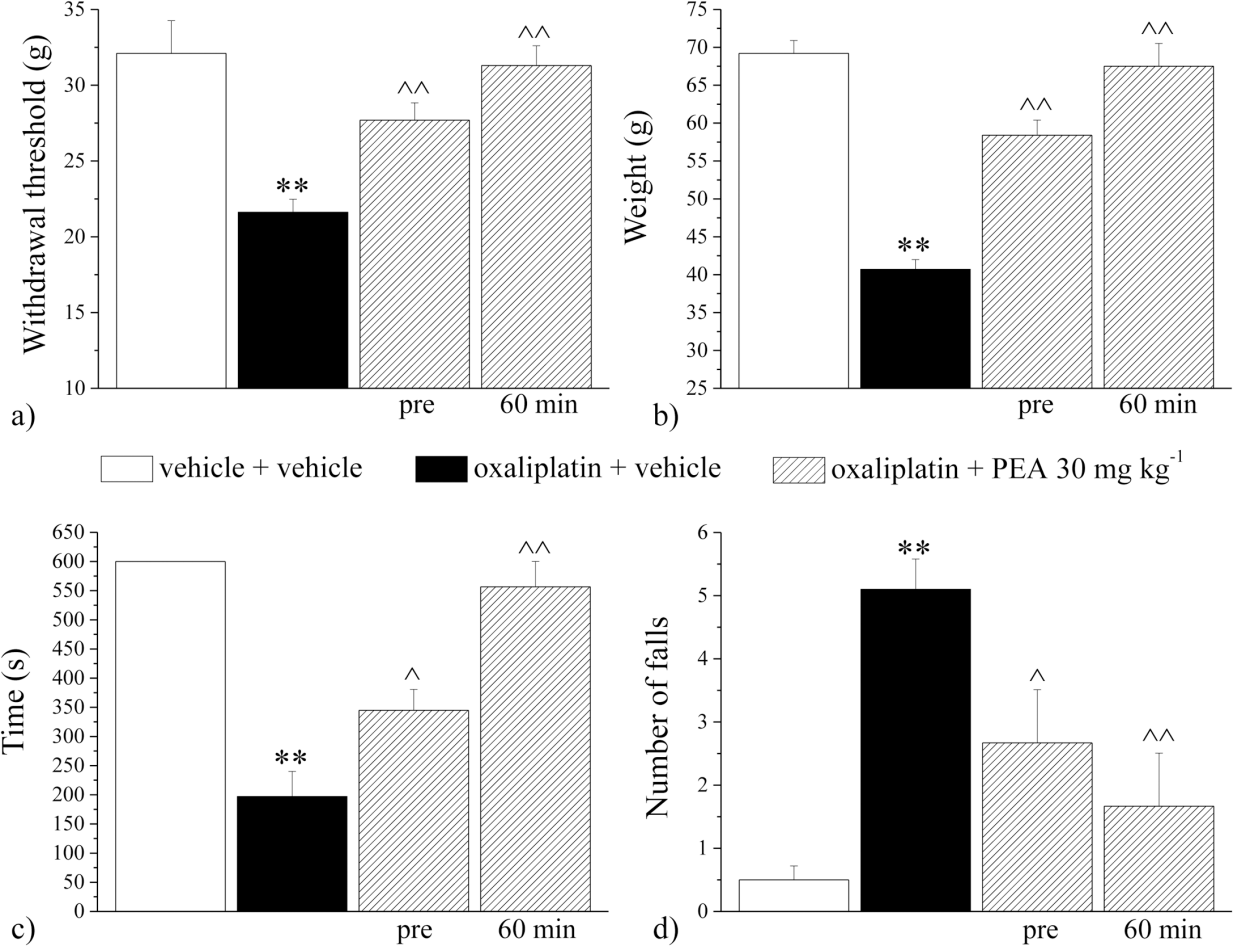
Behavioral measures. *Pain*: *mechanical non-noxious and noxious stimuli*. a) The Von Frey test was used to measure the pain threshold as a response evoked by a non-noxious stimulus. b) Paw-pressure test was used to measure sensitivity to a mechanical noxious stimulus. *Motor coordination*. The integrity of the animals’ motor coordination was assessed using a Rota-rod apparatus measuring c) the time spent to keep the balance and d) the number of falls, in 600 s. Animals were treated daily i.p. with 2.4 mg kg^-1^ oxaliplatin or vehicle. PEA (30 mg kg^-1^) was administered daily i.p. Behavioral evaluations were performed on day 21, 24h after treatment (pre) and 60 min after a new injection. Control animals were treated with vehicles. Each value represents the mean of 12 rats per group, performed in two different experimental sets. **P<0.01 versus vehicle + vehicle; ^P<0.05 and ^^P<0.01 versus oxaliplatin + vehicle.

Oxaliplatin administration also altered the sensitivity to mechanical stimuli (Fig 2A and 2B). As measured with the electronic Von Frey apparatus, the withdrawal threshold to the non-noxious mechanical stimulus was decreased in oxaliplatin-treated animals (day 21) from 32.1 ± 1.1 g, vehicle + vehicle, to 21.6 ± 1.1 g, oxaliplatin + vehicle (Fig 2A). On day 21, PEA (30 mg kg^-1^ injected daily from day 0 to day 20) prevented pain threshold alteration by 55% (pre). Sixty min after a further administration pain is fully reverted (Fig 2A; 60 min). The response to a noxious mechanical stimulus revealed mechanical hypersensitivity: the weight tolerated on the posterior paw, measured by the Paw-pressure test, significantly decreased from the control value of 69.2 ± 1.7 g (Fig 2B) to 40.5 ± 1.3 g for oxaliplatin-treated animals. PEA repeated treatment reduced oxaliplatin-induced hypersensitivity by about 62% (pre). In addition, when tested 60 min after a new injection of PEA (30 mg kg^-1^), the weight tolerated is comparable to that of control animals. On day 21, motor coordination was evaluated by Rota rod test measuring the walking time and the number of falls in 600 s. In comparison with control rats (time 600 s; number of falls 0.5 ± 0.2) oxaliplatin treated animals maintained the balance for 197.3 ± 43.2 s (Fig 2C) and fell down 5.1 ± 0.5 times (Fig 2D). On day 21, motor alteration was significantly relieved (40% and 53% for time and number of falls, respectively) by repeated administration of PEA (Fig 2C and 2D, pre). Sixty min after the new injection the relief increased to 89% and 75%, respectively (Fig 2C and 2D, 60 min).

### Effect of PEA on morphological and biomolecular derangement of the peripheral and central nervous system

**Fig 3:**
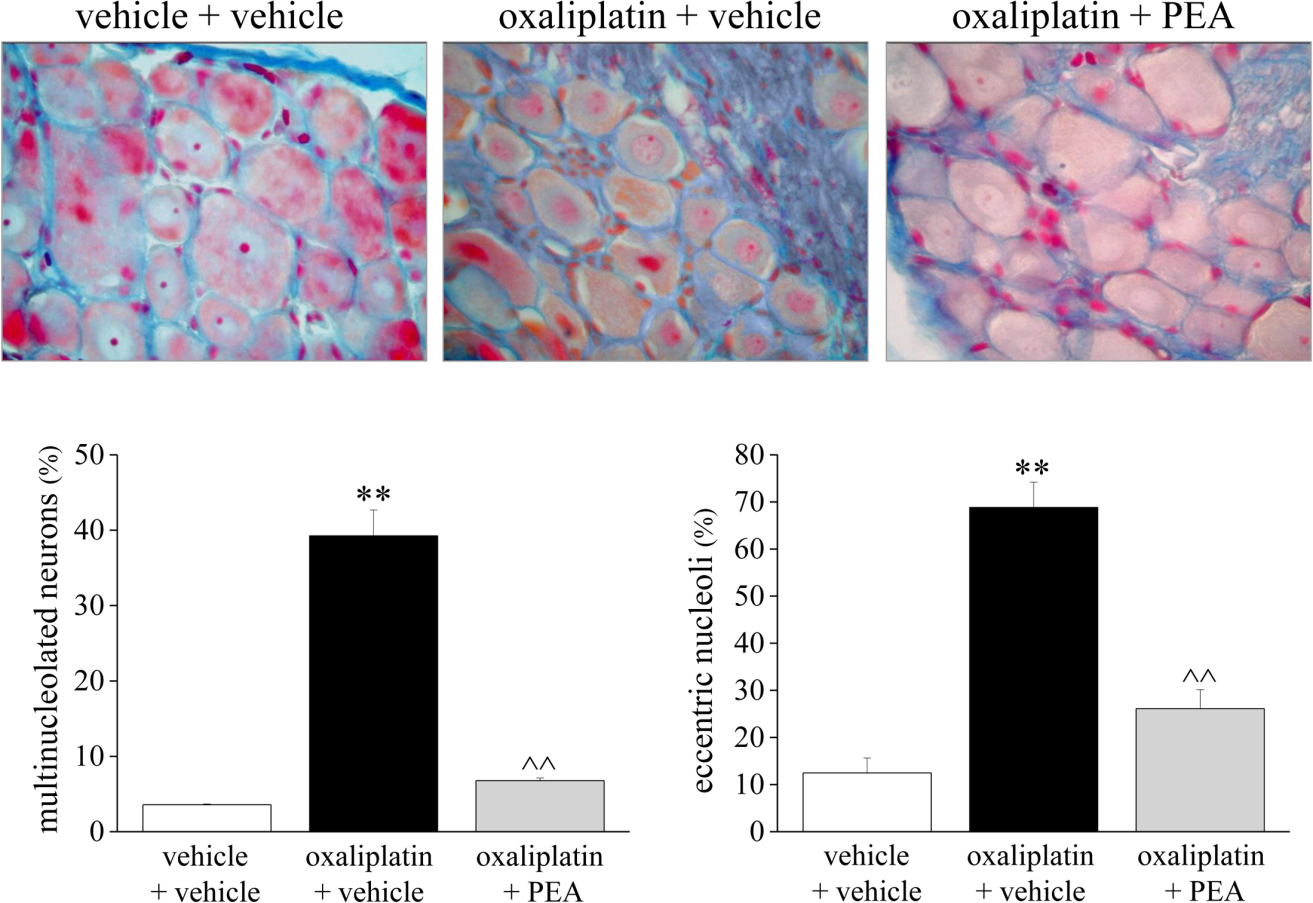
Morphological aspects of the peripheral nervous system. The protective effect of repeated administrations of PEA was evaluated on oxaliplatin-damaged DRGs on day 21. 5 μm DRG sections were stained by the Azan-Mallory method. Light micrographs (original magnification 20X) were analyzed by measuring the incidence of eccentric nucleoli and multinucleolated neurons. Each value represents the mean of 12 rats per group, performed in two different experimental sets. **P<0.01 versus vehicle + vehicle; ^^P<0.01 versus oxaliplatin + vehicle.

The histological determinations performed on lumbar DRGs from oxaliplatin-treated rats revealed characteristic damage illustrated in Fig 3. PEA exerted a significant protective effect by reducing the occurrence of multinucleolated neurons and the nucleolar eccentricity caused by oxaliplatin by about 90% and 76%, respectively. PEA also prevented the decrease of the somatic area of small and medium neurons highlighted in oxaliplatin-treated rats (Table 1).

**Table 1:** Morphometric determinations performed on the soma area of DRG Neurons.

	Soma area (m2)		
	small neurons	medium neurons	large neurons
	< 600 μm2	600–1200 μm2	> 1200 μm2
**vehicle + vehicle**	436.0 ± 6.8	966.9 ± 21.3	1346 ± 27.8
**oxaliplatin + vehicle**	358.6 ± 18.1**	825.2 ± 20.3**	1328.3 ± 23.7
**oxaliplatin + PEA**	401.2 ± 10.5^^	894.3 ± 18.5^^	1332.4 ± 26.1

**P<0.01 in comparison to vehicle + vehicle treated rats

^^P<0.01 vs oxaliplatin + vehicle group.

**Fig 4:**
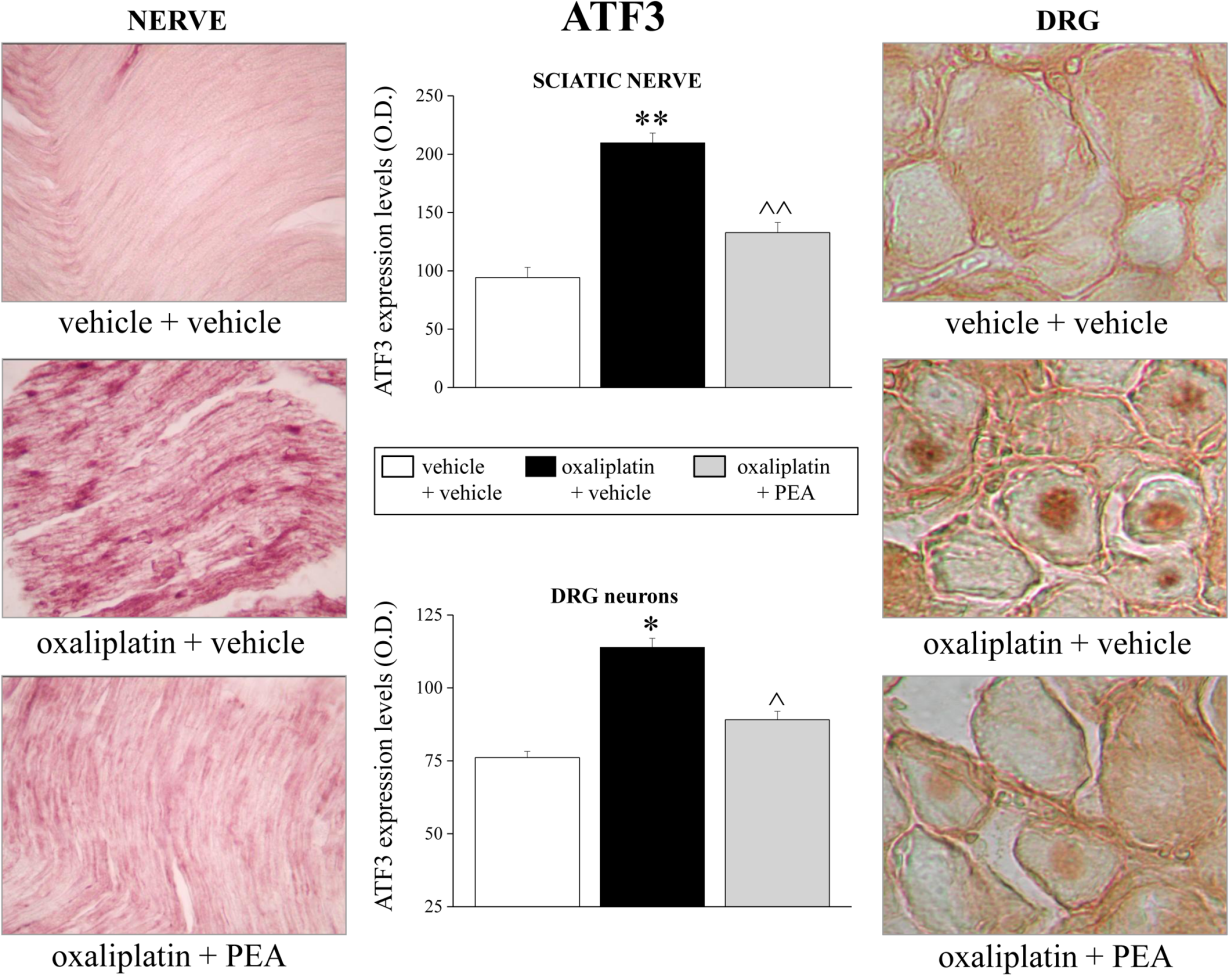
ATF3 expression levels in sciatic nerve and L4-L5 DRGs. The protective effect of PEA was evaluated on the peripheral nervous tissue of treated animals on day 21. A representative immunohistochemical staining for ATF3 in 10 μm longitudinal sciatic nerve sections is shown (original magnification 20X). Densitometric analysis was performed to obtain a quantitative measurement for sciatic nerve and DRG neurons. Each value represents the mean of 12 rats per group, performed in two different experimental sets. *P<0.05 and **P<0.01 versus vehicle + vehicle; ^P<0.05 and ^^P<0.01 versus oxaliplatin + vehicle.

To investigate the ATF3 expression profile in the sciatic nerve and lumbar 4–5 DRGs, immunostaining analyses were performed in comparable sections of tissue from all treatment groups. Fig 4 shows the significant ATF3 increase in both tissues after oxaliplatin treatment compared to vehicle + vehicle treated rats. This difference in ATF3 protein expression levels was drastically reduced in animals treated concurrently with oxaliplatin and PEA.

**Fig 5:**
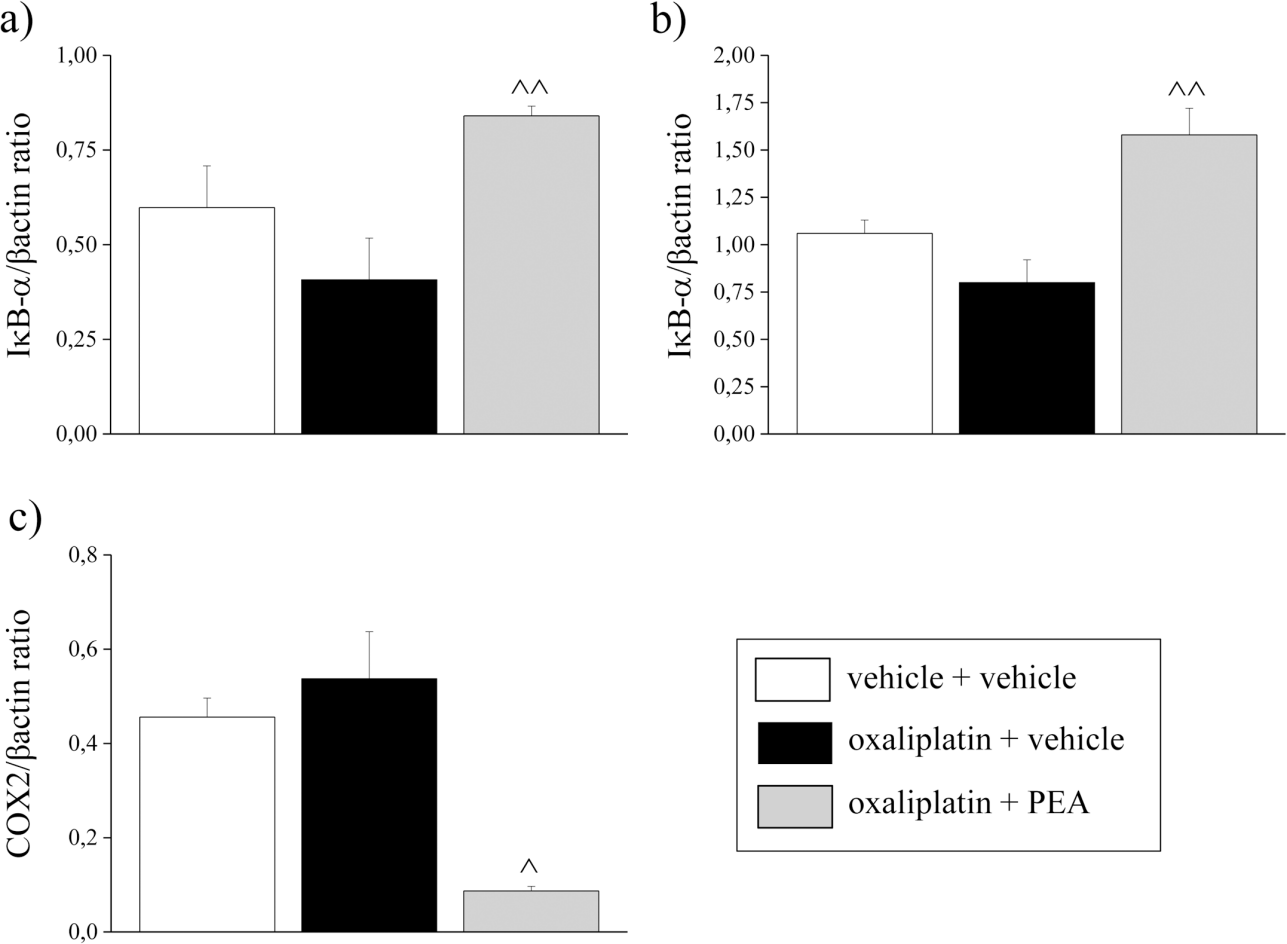
Inflammation-related mediators. On day 21, protein expression levels of IκBα were quantified by immunoblot in a) DRG and b) spinal cord; c) protein expression levels of COX2 were quantified by immunoblot in spinal cord Animals were treated daily i.p. with 2.4 mg kg^-1^ oxaliplatin or vehicle for 21 days. PEA (30 mg kg^-1^) was repeatedly administered i.p. (daily for 20 days starting from the first day of oxaliplatin administration). Control animals were treated with vehicles. Densitometric analysis is shown. β-actin normalization was performed for each sample. Each value represents the mean of 5 rats per group, performed in two different experimental sets. ^P<0.05 and ^^P<0.01 versus oxaliplatin + vehicle.

On day 21, IB- expression wasn’t modified by oxaliplatin treatment in DRGs and spinal cord, while PEA repeated treatment (Fig 5 and S1 Fig; oxaliplatin + PEA) was able to increase IB- expression by about 97% in both DRGs and spinal cord in comparison to oxaliplatin + vehicle group. Moreover, PEA was able to decrease COX2 expression in the spinal cord by about 87% in comparison to oxaliplatin + vehicle group (Fig 5 and S1 Fig).

### Effect of PEA treatment on NS neuron activity

**Fig 6:**
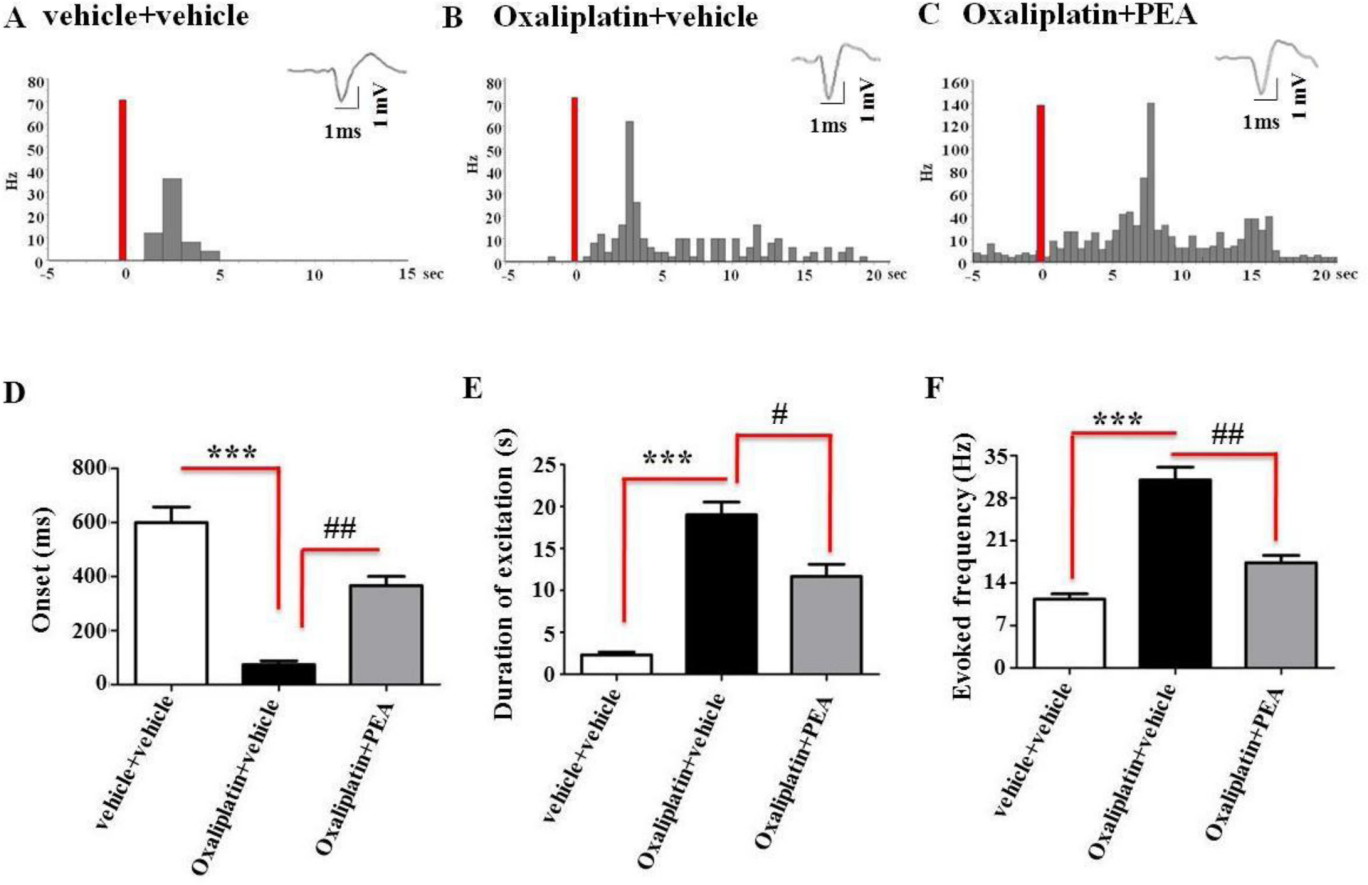
Electrophysiological recording of *NS neuron* activity. Representative peristimulus time histograms (PSTHs) show the responses of a single spinal NS neuron to a mechanical noxious stimulation (von Frey filaments 5.8N/20mm^2^ for 3sec) in vehicle (A), oxaliplatin (B) and oxaliplatin + PEA (C) treated rats on day 21 of treatment. The lower panels show the onset (E), the duration of excitation (F), and the frequency (G) of the evoked activity of NS neurons in the three groups. Each point represents the mean ± S.E.M of 2–3 neurons recorded for each animal of different groups of rats (n = 3–6). ***P<0.001 indicates significant differences versus vehicle + vehicle, ^#^P<0.05 and ^##^P<0.01 indicate significant differences versus oxaliplatin + PEA.

The results are based on NS neurons at a depth of 1–3 mm from the surface of the spinal cord. This cell population was characterized by a mean rate of spontaneous firing of 0.015 ± 0.002 Hz. Thus, only cells showing this pattern of basal firing were chosen for the recordings. The electrophysiological studies measured the onset of excitation (the time from the application of the stimulus artefact to the first evoked spike exceeding the average baseline value + 2 standard deviations), the frequency of the evoked excitatory responses and the duration of excitation (the period in ms of the increased firing activity which exceeds the average baseline value + 2 standard deviations). No change in the spontaneous and evoked activity of NS neurons were found in the control rats treated with vehicle (0.09 ± 0.002 Hz) (Fig 6) with respect to the naïve (not shown). In contrast, we observed an overall NS neuron hyper-excitability in oxaliplatin treated rats as compared to the control rats. In particular, we found a significant decrease in the onset of the evoked activity (115 ± 18 ms, P<0.001) and an increase in the duration (18 ± 1.8 s, P<0.001) and the frequency of the evoked activity (29.6 ± 2.3 Hz, P <0.001), n = 6, (Fig 6).

Repeated PEA treatment, significant reverted the induced-oxaliplatin changes in the spinal cord neuronal activity. In particular, PEA increased the onset (360 ± 18.36 ms, P<0.01) and reduced the frequency (16 ± 1.29 Hz, P<0.01) and the duration of the evoked activity (10 ± 0.9 s, P<0.05), n = 6 21 days after the treatment as compared to oxaliplatin-treated rats (Fig 6).

Representative peri-stimulus time histograms show the activity of a single NS neuron in control, oxaliplatin and oxaliplatin + PEA treated rats on day 21 (Fig 6A, 6B and 6C).

### Effect of PEA treatment on glial cell activation profile

**Fig 7:**
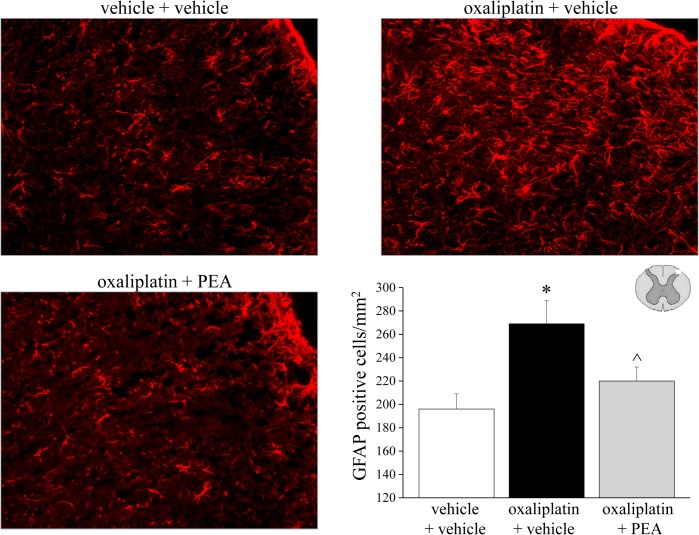
Glial activation profile in the spinal cord. *Astrocytes*. The effect of repeated treatment with PEA (30 mg kg^-1^ daily i.p.) was evaluated in oxaliplatin-treated rats on day 21. The number of GFAP-positive cells was measured in the dorsal horn of the spinal cord. Images (original magnification 20X) of sections of lumbar spinal cord of oxaliplatin-treated animals (oxaliplatin + vehicle) are reproduced in comparison with control (vehicle + vehicle). Representative immunohistochemical staining after PEA treatments is shown (20X). Each value represents the mean of 12 rats per group, performed in two different experimental sets. *P<0.05 versus vehicle + vehicle; ^P<0.05 versus oxaliplatin + vehicle.

**Fig 8:**
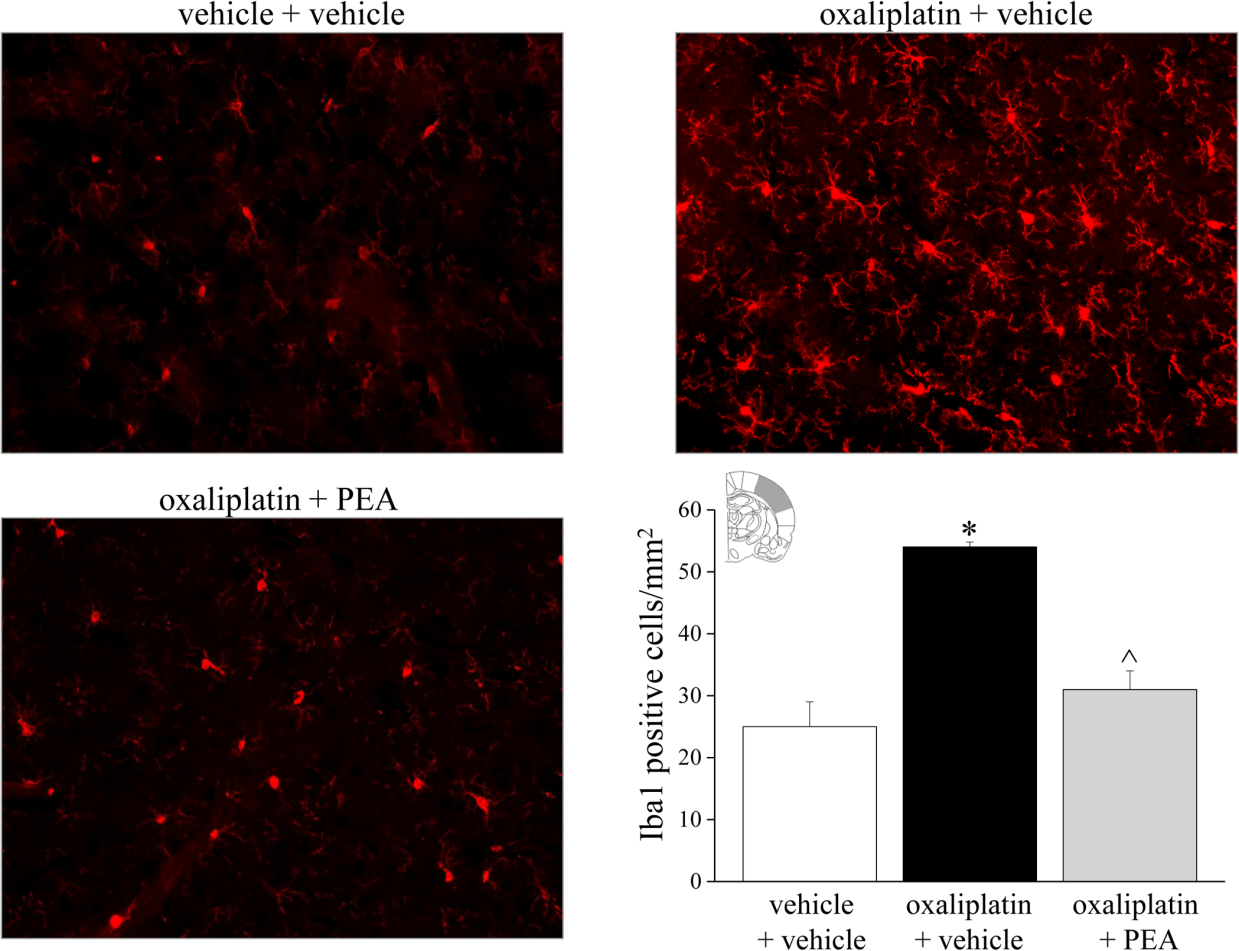
Glial activation profile in the brain cortex. *Microglia*. The effect of repeated treatment with PEA (30 mg kg^-1^ daily i.p.) was evaluated in oxaliplatin-treated rats on day 21. The number of Iba1-positive cells were measured in the somatosensory area 1. Representative immunohistochemical staining (20X) and quantitative measurements are shown. Each value represents the mean of 12 rats per group, performed in two different experimental sets. *P<0.05 versus vehicle + vehicle; ^P<0.05 versus oxaliplatin + vehicle.

**Fig 9:**
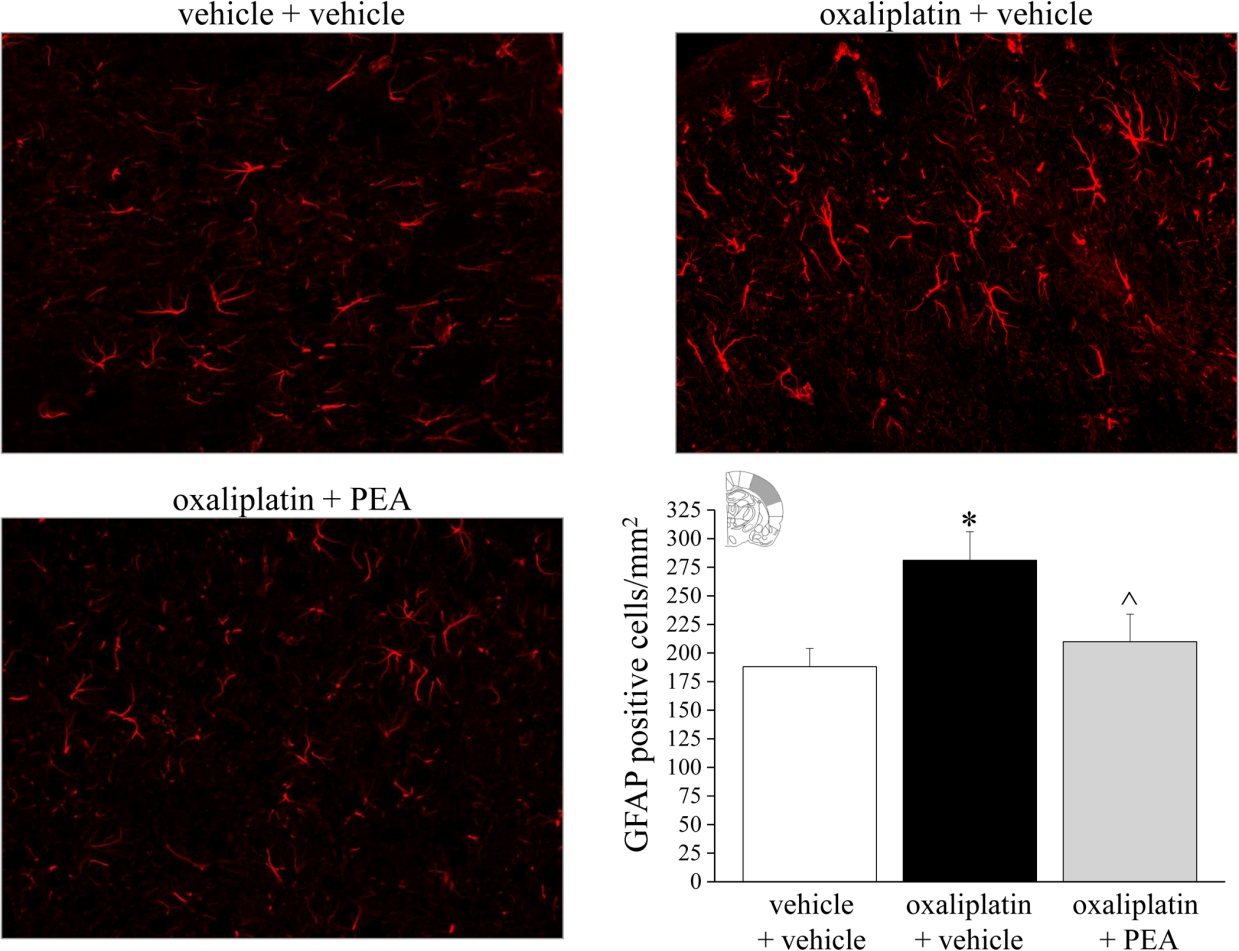
Glial activation profile in the brain cortex. *Astrocytes*. The effect of repeated treatment with PEA (30 mg kg^-1^ daily i.p.) was evaluated in oxaliplatin-treated rats on day 21. The number of GFAP-positive cells were measured in the somatosensory area 1. Representative immunohistochemical staining (20X) and quantitative measurements are shown. Each value represents the mean of 12 rats per group, performed in two different experimental sets. *P<0.05 versus vehicle + vehicle; ^P<0.05 versus oxaliplatin + vehicle.

The central nervous system was analyzed to assess glial cells reorganization after PEA treatment. In the spinal cord, repeated oxaliplatin injections (day 21) induced an increase in GFAP staining (Fig 7), astrocyte density increased over the entire surface of the spinal cord, particularly in the superficial laminae. PEA prevented the increase in the number of the dorsal horn GFAP-positive cells by 66%. As depicted in S2 Fig, the microglial cell number in the spinal cord (labeled immunohistochemically with antibodies against Iba1) was not altered on day 21 of the oxaliplatin protocol. On the contrary, at the supraspinal level, in somatosensory area 1 (S1) oxaliplatin increased the number of Iba1-positive cells (Fig 8) as well as astrocyte density (Fig 9). PEA fully prevented the increase both in microglia and astrocyte cell number (Figs 8 and 9).

### Effect of PEA on HT-29 cells in the presence of oxaliplatin

In order to evaluate the potential interaction between PEA treatment and the therapeutic property of oxaliplatin, we measured the viability of the human colon cancer cell line HT-29. Table 2 shows the lack of influence by PEA on the concentration-dependent (0.3–100 μM) oxaliplatin lethal effect after 24 and 48h incubation.

**Table 2:** HT-29 cell viability after 24 and 48h incubation.

	Cell viability %			
	24h incubation		48h incubation	
Oxaliplatin concentration	Control	PEA	Control	PEA
(μM)		(10 μM)		(10μM)
0	100.0 ± 3.2	100.0 ± 3.2	100.0 ± 2.3	100.0 ± 2.3
0.3	98.9 ± 5.5	98.8 ± 5.3	92.3 ± 3.4	91.2 ± 4.2
1	98.4 ± 4.2	95.2 ± 4.8	92.9 ± 0.3	88.6 ± 5.1
3	94.4 ± 2.1	91.8 ± 3.2	91.0 ± 4.4	86.2 ± 6.3
10	92.2 ± 2.1*	87.2 ± 6.5*	83.5 ± 2.3**	84.8 ± 4.7**
30	86.5 ± 2.3**	83.5 ± 2.6**	83.4 ± 5.1**	79.4 ± 4.5**
100	74.9 ± 2.4**	71.7 ± 1.4**	57.4 ± 4.6**	56.5 ± 1.1**

HT-29 cells were treated with increasing concentrations of oxaliplatin (1–100 μM) in the presence or in the absence of PEA (10 μM). Incubation was allowed for 24h or 48h. Cell viability was measured by MTT assay. Control condition was arbitrarily set as 100% and values are expressed as the mean ± S.E.M. of three experiments.

*P<0.05

**P<0.01 in comparison to control (oxaliplatin 0 μM).

## Discussion

Painful chemotherapy-induced neuropathy can persist from months to years beyond chemotherapy completion, causing significant challenges for cancer survivors due to negative influence on function and quality of life [49], [50], [51], [52], [53]. Neurotoxicity may results in chemotherapy dose reductions or early discontinuation. The overall incidence of CIPN is estimated to be approximately 38% in patients treated with multiple agents [54]. Chemotherapy combinations with higher incidences include those that involve platinum drugs, vinca alkaloids, bortezomib, and/or taxanes [55]. The 2014 clinical practical guideline from the American Society of Clinical Oncology states that there are no agents recommended for the prevention of chemotherapy-induced neuropathic pain. High-quality, consistent evidence are insufficient, the best available data support a moderate recommendation for treatment with duloxetine. Tricyclic antidepressants, gabapentin, or topical gel containing baclofen, amitriptyline, and ketamine may be offered on the basis of data supporting their utility in other neuropathic pain conditions given the limited other chemotherapy-induced neuropathic pain treatment options [56]. In particular for oxaliplatin neurotoxicity, the most recent therapeutic advancement is the combination of intermittent oxaliplatin administration and the use of concurrent calcium and magnesium salts [57].

In the present research the efficacy of PEA, after acute or repeated treatment, was highlighted in a preclinical model of oxaliplatin-induced neuropathy. PEA significantly reduced oxaliplatin-dependent pain, when evaluated as an increase upon suprathreshold stimulation (hyperalgesia-related measurement) or as a decrease in pain threshold (allodynia-related measurement). Furthermore, an improvement in motor coordination is evidenced. The pain relief efficacy is maintained after repeated treatment excluding tolerance development. The repeated administration protocol allows to maintain a controlled pain threshold, sensitive to the additive effect of a new administration. Noteworthy, PEA is not analgesic since it does not modify the physiological pain threshold of control animals. The property to electively normalize conditions of hypersensitivity is highlighted.

The pain reliever effect of PEA repeated administrations is accompanied by a protective effect from the alterations of the peripheral and central nervous system evoked by oxaliplatin suggesting a disease modifying effect. In line with previous evidence [32], [37], [39], [58], [59], detailed morphological analysis demonstrates that DRGs are a primary target for oxaliplatin neurotoxicity. PEA prevents morphological derangements in DRGs as well as the significant increase in ATF3 expression, a member of the ATF3/cAMP-responsive element binding protein (CREB) family [60] both in the DRG neurons and in the Schwann cells of the peripheral nerve of oxaliplatin-treated-rats. The protective effects of PEA result in functional normalization highlighted by the electrophysiological measurements performed in the spinal cord. Renn and co-workers [34] observed an increased activity of wide dynamic range neurons in the spinal dorsal horn of oxaliplatin-treated mice, the present data evidence an overall dorsal horn nociceptive-specific neuron hyper-excitability. Consistently with the behavioral data, PEA i.p. administered reduces the oxaliplatin-dependent changes in the spinal cord neuronal activity. Accordingly, Luongo et al. [61] described the reduction of the formalin-dependent neuronal activity induced by PEA when locally applied onto the dorsal horn.

Besides the neuronal damage and the neuronal maladaptive plasticity, glial cells have recently been recognized as a powerful modulator of pain. In models of trauma-induced neuropathy, microglia appear to exert a key role in the initial phases of neuropathic pain whereas astrocytes may be involved in its maintenance [62], [63]. In addition, glial inhibitors have been described as pain relievers and glial cells are emerging as a new target for drug development [64], [65]. Despite the oxaliplatin limited ability to cross the blood brain barrier [66], [67], we previously described the glial activation induced by oxaliplatin in spinal cord and brain areas, differently according to cell type, anatomical region, and treatment time-points [37]. The increased cell density of microglia and astrocyte is strongly related to pain hypersensitivity since the glial inhibitor minocycline and fluorocitrate fully prevent oxaliplatin-evoked pain [68]. The present results reveal an inhibitory effect of PEA on microglia and astrocytes in the dorsal horn of the spinal cord and in brain S1 with decreasing in the number of both cell types. Glial cells are a target for PEA [25], accordingly PEA normalized spinal microglia and astrocyte activation in the rat model of inflammatory pain induced by formalin [61] as well as after spinal cord trauma in mice [69]. Moreover, we recently showed the property of PEA to attenuate morphine tolerance by a glia-mediated mechanism [70]. Interestingly, PEA seems to be able to modulate glial cells instead to act as a general depressor of glial functions [71]. The homeostatic properties of PEA may allow the inhibition of glial hyper-reactivity preserving neuroprotection (differently from the glial blockers minocycline and fluorocitrate [68]), a housekeeping role of these cells [72].

To this framework participates also the anti-inflammatory effects of PEA described in the present results by increased IB- expression in both DRGs and spinal cord and decreased COX-2 in the spinal cord. On the other hand, neither IκB-α, the protein that inactivates NF-κB preventing its translocation to the nucleus and the transcription activation of κB-dependent genes [73], nor COX-2, described together with its end product prostaglandin E2 involved in neuropathic pain development [74], are significantly modified by oxaliplatin. This evidence confirms the limited role of inflammation in oxaliplatin neurotoxicity, as previously demonstrated by a morphological analysis of the nervous system [37]. The predominance of the neuropathic component in oxaliplatin-induced pain is suggested.

A complex panel of pharmacodynamic targets may account the present combination of effects on oxaliplatin-induced neuropathy. PEA increases the antioxidant defense reducing oxidative stress [75], a characteristic feature of oxaliplatin neurotoxicity strongly related to pain [76]. Moreover, the activation of the αsubtype of the peroxisome proliferator-activated receptors (PPAR-α) has a pivotal role in PEA-mediated pain relief (after single [21] and repeated [20] administrations) as well as in the neurorestorative properties after traumatic peripheral nerve injury [21]. PPAR-α participates also to the PEA modulation of microglial cells [77]. PEA, through PPAR-α, induces allopregnanolone synthesis in astrocytes [78] and in the rat spinal cord involving the *de novo* neurosteroid synthesis in the modulation of pain behavior [79].

Nevertheless, PEA is known to mimic several endocannabinoid-driven actions even though PEA does not bind CB1, CB2, and abn-CBD receptors [80]. An “entourage effect hypothesis” has also been formulated on the basis of an activity enhancement of other endogenous compounds (e.g. the endocannabinoid anandamide; Calignano et al., [19], by potentiating their affinity for a receptor or by inhibiting their metabolic degradation [81], PEA may indirectly stimulate the transient receptor potential vanilloid type 1 (TRPV1) and the cannabinoid receptors [25]. Interestingly, an alteration of spinal endocannabinoid (anandamide and 2-arachidonoylglycerol) levels was demonstrated after cisplatin treatment in rat whereas the inhibition of endocannabinoid hydrolysis alleviates chemotherapy-induced mechanical and cold allodynia [82]. Anandamide, acutely injected, is effective against cisplatin-induced pain by a CB1-mediated mechanism [83].

Finally, it is important to highlight the absence of interaction between PEA and the lethal effect exerted by oxaliplatin on the human colon cancer cells HT-29, accordingly with the anti-proliferative properties previously assessed in vitro [84], [85]. Moreover, the good tolerability of PEA is described in several clinical studies [24], [86], [87].

## Conclusions

PEA is able to control pain and prevent alterations of both the peripheral and central nervous system induced in rat by oxaliplatin. Pea may offer a dual protective approach against etiological factors and resulting maladaptative plasticity.

## Supporting Information

S1 Fig Inflammation-related mediators. On day 21, protein expression levels of IκBα were quantified by immunoblot in a) DRG and b) spinal cord; c) protein expression levels of COX2 were quantified by immunoblot in spinal cord. Animals were treated daily i.p. with 2.4 mg kg^-1^ oxaliplatin or vehicle for 21 days. PEA (30 mg kg^-1^) was repeatedly administered i.p. (daily for 20 days starting from the first day of oxaliplatin administration). Control animals were treated with vehicles. Representative blot of lysates are shown.
(TIF)

S2 Fig Glial activation profile in the spinal cord. ***Microglia*.** The effect of repeated treatment with PEA (30 mg kg^-1^ daily i.p.) was evaluated in oxaliplatin-treated rats on day 21. The number of Iba1-positive cells was measured in the dorsal horn of the spinal cord. Images (original magnification 20X) of sections of lumbar spinal cord of oxaliplatin-treated animals (oxaliplatin + vehicle) are reproduced in comparison with control (vehicle + vehicle). Representative immunohistochemical staining after PEA treatments is shown (20X). Each value represents the mean of 12 rats per group, performed in two different experimental sets. *P<0.01 versus vehicle + vehicle; ^P<0.01 versus oxaliplatin + vehicle.
(TIF)

S1 Table Evaluation of PEA effect on the normal pain threshold. PEA (30 mg kg^-1^ i.p.) was acutely administered at vehicle-treated animals on day 21 to evaluate the effect on nociceptive threshold of normal (non-hypersensitive) animals. The response to thermal stimuli was evaluated both in the Hot and Cold Plate tests measuring the latency to pain-related behavior (lifting or licking of the paw). The response to a mechanical stimulus was evaluated in the Paw pressure test measuring the weight tolerated on the posterior paw. Each value represents the mean of 12 rats per group, performed in two different experimental sets.
(DOCX)
