# Supplementary figures and images for: Antineuropathic Profile of N-Palmitoylethanolamine in a Rat Model of Oxaliplatin-Induced Neurotoxicity
Source: PLoS One. 2015 Jun 3;10(6):e0128080. doi: 10.1371/journal.pone.0128080 (PMC4454493; doi:10.1371/journal.pone.0128080)

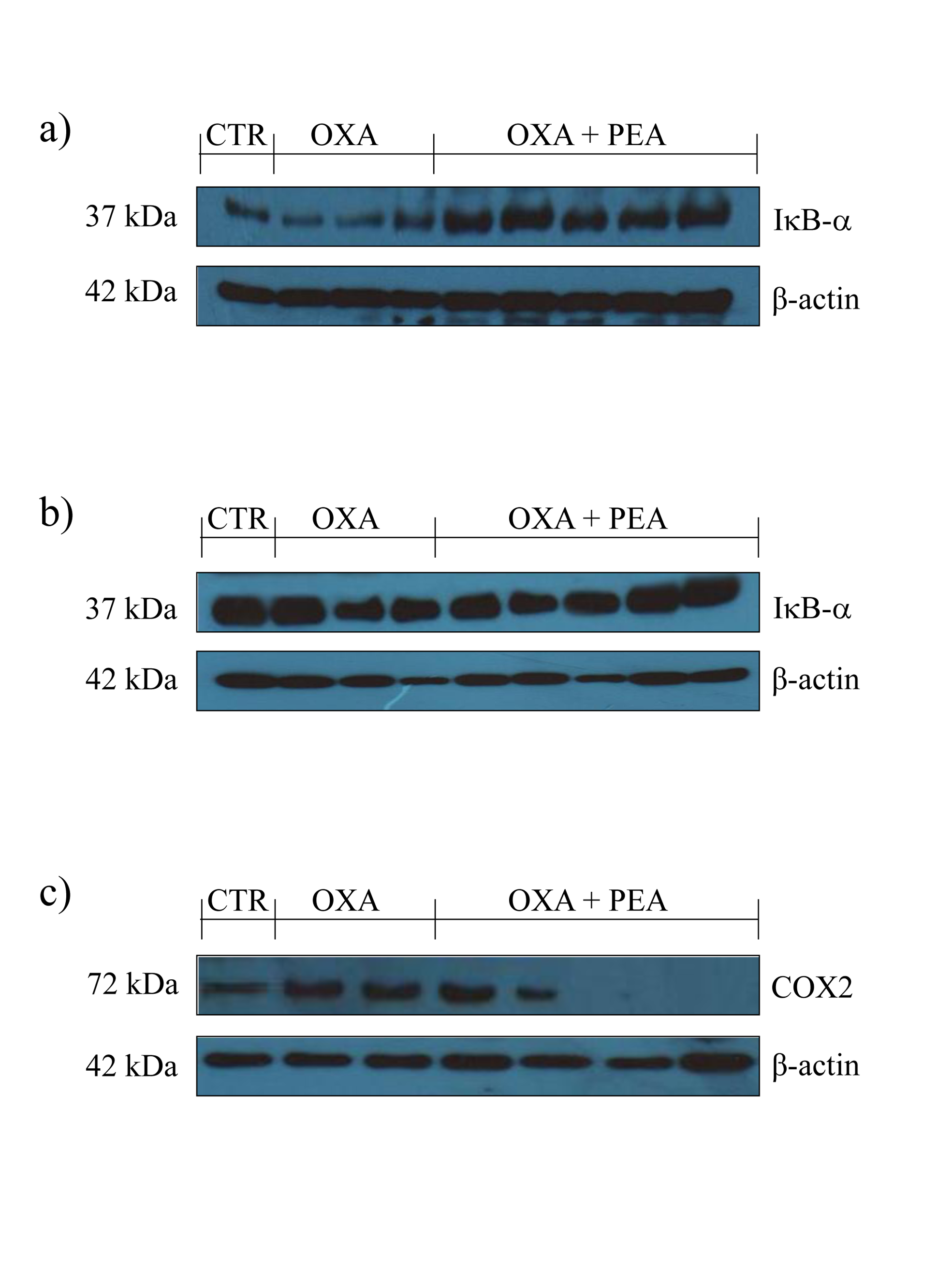

Supplement: S1 Fig — On day 21, protein expression levels of IκBα were quantified by immunoblot in a) DRG and b) spinal cord; c) protein expression levels of COX2 were quantified by immunoblot in spinal cord. Animals were treated daily i.p. with 2.4 mg kg-1 oxaliplatin or vehicle for 21 days. PEA (30 mg kg-1) was repeatedly administered i.p. (daily for 20 days starting from the first day of oxaliplatin administration). Control animals were treated with vehicles. Representative blot of lysates are shown. (TIF) [file pone.0128080.s001.tif]

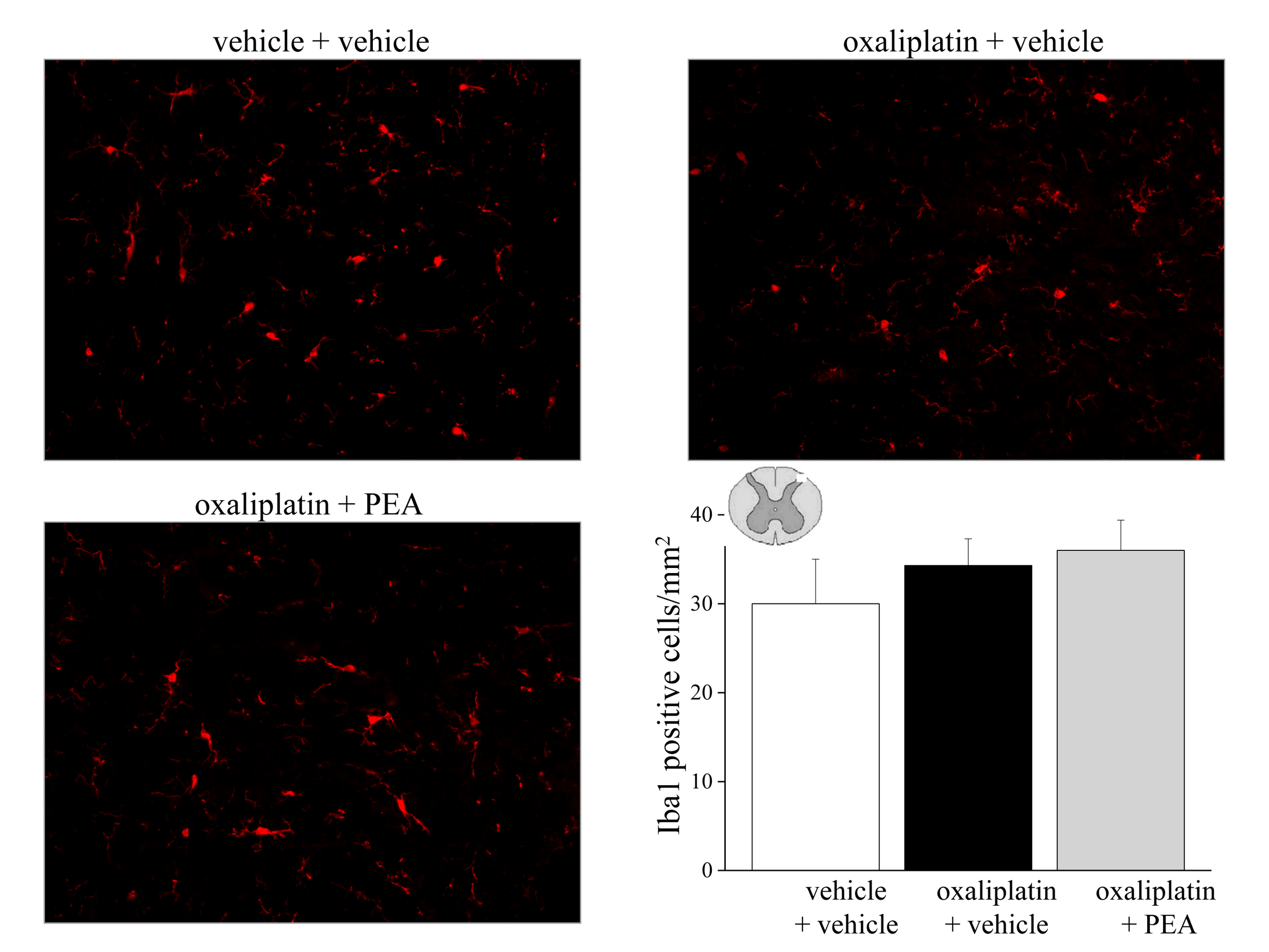

Supplement: S2 Fig — Microglia. The effect of repeated treatment with PEA (30 mg kg-1 daily i.p.) was evaluated in oxaliplatin-treated rats on day 21. The number of Iba1-positive cells was measured in the dorsal horn of the spinal cord. Images (original magnification 20X) of sections of lumbar spinal cord of oxaliplatin-treated animals (oxaliplatin + vehicle) are reproduced in comparison with control (vehicle + vehicle). Representative immunohistochemical staining after PEA treatments is shown (20X). Each value represents the mean of 12 rats per group, performed in two different experimental sets. *P<0.01 versus vehicle + vehicle; ^P<0.01 versus oxaliplatin + vehicle. (TIF) [file pone.0128080.s002.tif]
