# Supplementary material for: Antineuropathic Profile of N-Palmitoylethanolamine in a Rat Model of Oxaliplatin-Induced Neurotoxicity
Source: PLoS One. 2015 Jun 3;10(6):e0128080. doi: 10.1371/journal.pone.0128080 (PMC4454493; doi:10.1371/journal.pone.0128080)
Supplement: S1 Table — PEA (30 mg kg-1 i.p.) was acutely administered at vehicle-treated animals on day 21 to evaluate the effect on nociceptive threshold of normal (non-hypersensitive) animals. The response to thermal stimuli was evaluated both in the Hot and Cold Plate tests measuring the latency to pain-related behavior (lifting or licking of the paw). The response to a mechanical stimulus was evaluated in the Paw pressure test measuring the weight tolerated on the posterior paw. Each value represents the mean of 12 rats per group, performed in two different experimental sets. (DOCX) [file pone.0128080.s003.docx]

**S1 Table. Evaluation of PEA effect on the normal pain threshold**

|  | **PEA (30 mg kg^-1^ i.p.)** | | | | |
| --- | --- | --- | --- | --- | --- |
|  |  | **Time after treatment (min)** | | | |
|  | ***pretest*** | ***15*** | ***30*** | ***45*** | ***60*** |
| **Cold Plate**  (Licking latency, s) | 20.6 ± 0.5 | 19.4 ± 0.3 | 21.4 ± 0.4 | 22.6 ± 0.4 | 20.3 ± 0.6 |
| **Hot plate**  (Licking latency, s) | 13.2 ± 0.45 | 15.0 ± 0.4 | 15.8 ± 0.6 | 14.4 ± 0.2 | 14.8 ± 0.6 |
| **Paw pressure**  (Weight, g) | 65.0 ± 1.4 | 70.8 ± 3.7 | 62.5 ± 1.6 | 63.4 ± 2.5 | 64.6 ± 2.1 |

PEA (30 mg kg^-1^ i.p.) was acutely administered at vehicle-treated animals on day 21 to evaluate the effect on nociceptive threshold of normal (non-hypersensitive) animals. The response to thermal stimuli was evaluated both in the Hot and Cold Plate tests measuring the latency to pain-related behavior (lifting or licking of the paw). The response to a mechanical stimulus was evaluated in the Paw pressure test measuring the weight tolerated on the posterior paw. Each value represents the mean of 12 rats per group, performed in two different experimental sets.
